# Assessment of Paranasal Sinus Pathology in Patients Presenting for Brain MRI as Referred From General Practice or Neurologist Physicians

**DOI:** 10.7759/cureus.30487

**Published:** 2022-10-19

**Authors:** Ziyad A Almushayti

**Affiliations:** 1 Department of Radiology, College of Medicine, Qassim University, Buraydah, SAU

**Keywords:** opacification, retention cyst, mucosal thickening, mri, paranasal sinuses pathology

## Abstract

Objective

The aim of this study is to assess the incidence and kinds of paranasal sinus pathology in patients presenting for brain MRI as referred from general practice or neurologist physicians in Qassim region, Saudi Arabia.

Methods

This cross-sectional study included 210 patients with different neurological indications and presentations who underwent an MRI of the brain and were evaluated by the consultant radiologist for the presence of any paranasal sinuses pathologies such as mucosal thickening, retention cyst, opacification, and ostiomeatal complex obliteration. The analysis was carried out using the chi-square test.

Results

A total of 210 cases underwent brain MRI to evaluate clinically suspected brain disorders; there were 138 (65.7 %) males and 72 (34.3%) females. Mucosal thickening was the commonest pathological finding (111 cases, 52.9%). retention cysts were present in around 14.3%, meaning 30 cases, and partial opacification was present in around 10%, representing 21 cases. Total opacification was present in three (1.4%) cases. Also, ostiomeatal complex obliteration was present in three (1.4%) cases. Paranasal pathological findings were more in those aged ≤ 35 years, with 75 (35.7%) cases. mucosal thickening, partial opacification, total opacification, and ostiomeatal complex obliteration were more in males, whereas retention cysts were equal in both genders.

Conclusion

Pathological paranasal sinuses findings commonly occur at brain MRI during neurological disorder evaluation. These findings are usually benign, and mucosal thickening is the commonest. The majority of the pathological findings were observed in those aged ≤ 35 years and in the male gender. Detecting pathological findings in paranasal sinuses helps diagnose lesions unrelated to the suspected neurological disease.

## Introduction

Magnetic resonance imaging (MRI) is a modality of choice for evaluating various brain pathologies due to innate differences between different tissue structures [1]. Paranasal sinus abnormalities are described during brain MRI performed to determine sinus-associated abnormalities [2]. A wide range of sinus abnormalities can be visualized during brain MRI scanning, which gives the advantage of cross-sectional imaging [3,4]. These days, there is an increase in the frequency of brain imaging, including the paranasal sinuses [2]. Abnormalities such as mucosal thickening, retention cyst, and opacification are commonly found in the paranasal sinuses [5]. Although computed tomography (CT) is commonly used to diagnose paranasal sinus conditions, it is not always the best choice for assessing soft tissue masses and cysts. Due to the high diagnostic value of MRI, it has been justified for the treatment of patients with complicated fungal infections and other conditions [6]. The use of MRI for the evaluation of various anatomical structures in the sinonasal spaces has many advantages, such as assessment of anosmia, diagnosis of benign lesions, and treatment of complicated sinonasal malignancies [4]. Incidental abnormalities of the paranasal sinuses are common findings in different imaging modalities [7]. MRI can be used as an adjunct to CT for the assessment of ostiomeatal complex and sinus conditions. It can help radiologists make more accurate differential diagnoses and improve the clinical management of their patients [8]. Due to the increasing use of MRI to diagnose brain lesions, the interpretation of these images can help identify potential abnormalities in the paranasal cavity, ostiomeatal complex, and nasal cavity. This can help facilitate the early management of these conditions [9,10]. Having the necessary knowledge about the prevalence of these conditions can help lead to the early diagnosis and prompt treatment of paranasal sinus conditions [11]. Therefore, this study aims to investigate the prevalence and kinds of sinus pathology in patients undergoing MRI of the brain in the Qassim region, Buraydah, Saudi Arabia.

## Materials and methods

Case selection

A cross-sectional study was performed randomly on 210 patients, of which 138 (65.7%) were males and 72 (34.3%) were females, with ages ranging from 20 to 71 years old distributed as ≤35 years, 36-50 years, and above 50 years. The study was conducted in the Qassim region. Including patients who underwent a brain MRI for different neurological indications and presentations between September 2021 and June 2022, the majority of patients had complaints of headaches. Patients with a history of malignancy or trauma were excluded to avoid metastatic deposition or extension of the trauma to the paranasal sinuses. A power analysis was performed with alpha at 5% and beta at 20%. In this calculation, the power of the study sample size was estimated to be 80%. Oral and written informed consent was obtained from participants and approved by the Committee of Health Research Ethics, Deanship of Scientific Research, Qassim University (approval number 21-23-03).

Image analysis

**Figure 1:**
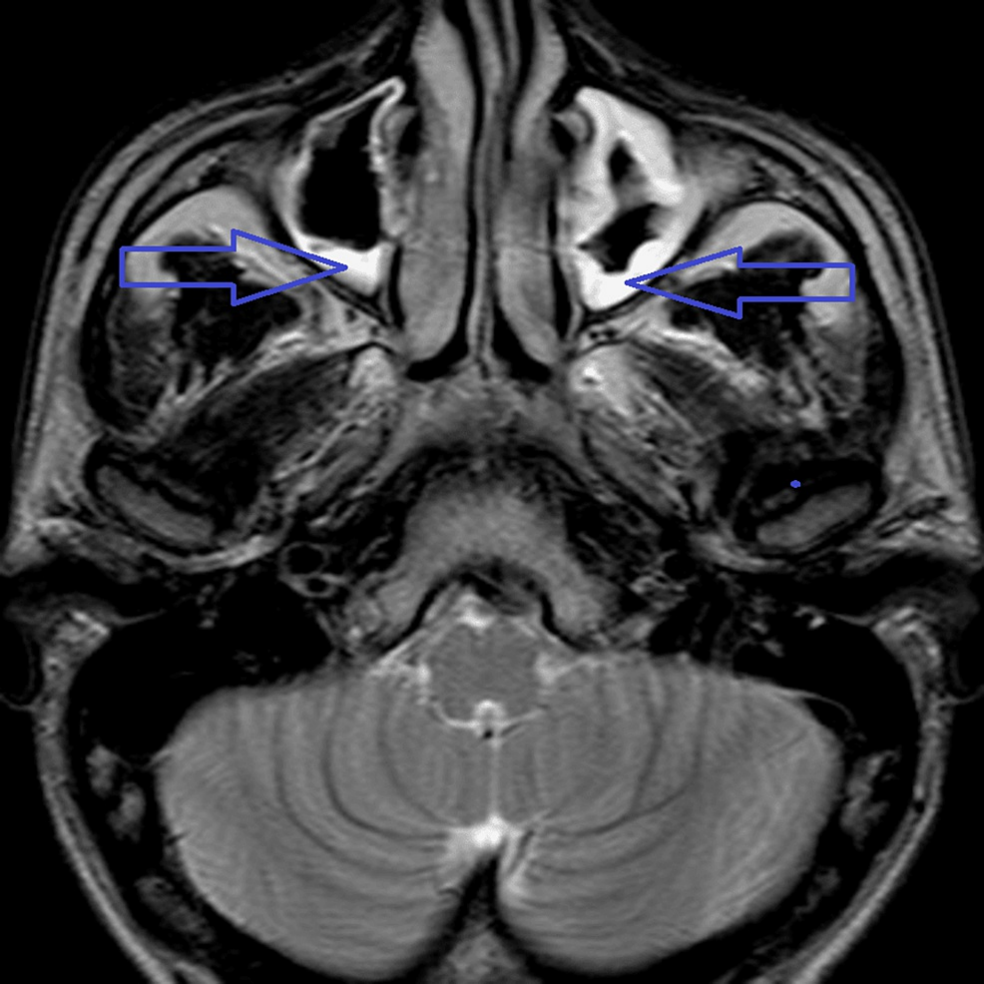
Axial T2-weighted image reveals mucosal thickening of both maxillary sinuses (arrows).

**Figure 2:**
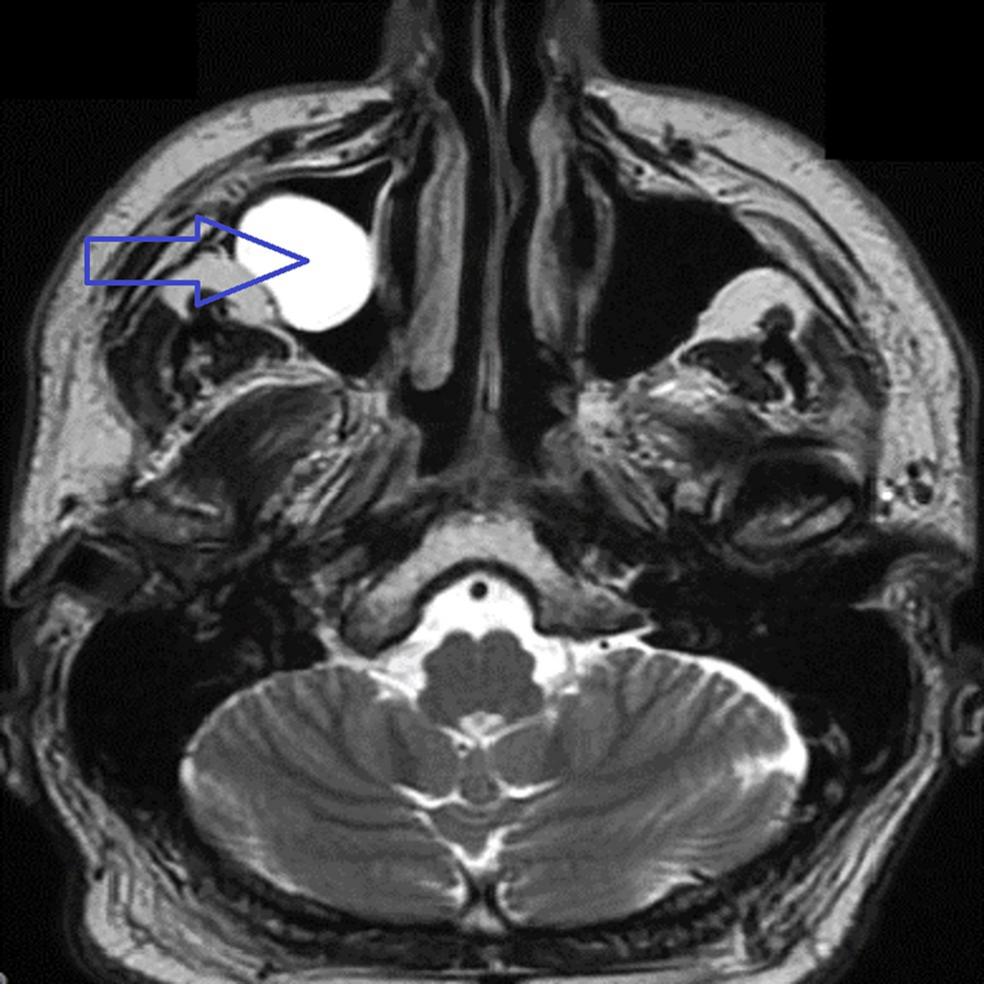
Axial T2-weighted image reveals retention cyst in the right maxillary sinus (arrow).

A consultant radiologist evaluated the MRI images for the presence of any pathological findings in the sinuses. The definition of pathological findings is the presence of any abnormality in the paranasal sinuses unrelated to the examination purpose. Many pathological findings were observed, including mucosal thickening, retention cyst, opacification, and ostiomeatal complex obliteration (Figures 1, 2). The study used patient demographic data and classified them according to gender and age groups as follows: ≤ 35 years, 36-50 years, and older than 50 years.

MRI parameters

The examinations were carried out on MRI machine using the same protocol; T2-weighted image (T2WI), fluid-attenuated inversion recovery (FLAIR), and diffusion-weighted imaging (DWI) sequences were obtained in the axial planes. Also, T2WI in the sagittal and coronal plane T1-weighted image (T1WI) sequences were performed. The MRI sequence parameters are given in Table 1.

**Table 1:** MRI sequence parameters. B-value, a factor that reflects the strength and timing of the gradients used to generate diffusion-weighted images; TI, inversion time in milliseconds; NEX, number of excitations; Gap, gaps between slices; FOV, the field of view in millimeters; PH DIR, phase direction; FLIP, flip angle; THK, slice thickness in millimeters; TE, echo time in milliseconds; TR, repetition time in milliseconds; FLAIR, fluid-attenuated inversion recovery; DWI, diffusion-weighted imaging; R, right; L, left; A, anterior; P, posterior

B-value	TI	NEX (average)	Gap	FOV	Matrix size	PH DIR	FLIP	THK	TE	TR	Sequence
		2	10%	230	320x320	R>L	150	5	100-120	3000-4000	T2 axial
	2500	2	10%	230	320x320	R>L	130	5	110	7000-9000	FLAIR axial
		2	10%	230	320x320	R>L	90	5	15-25	400-600	T1 coronal
		2	10%	230	320x320	A>P	150	5	100-120	3000-4000	T2 sagittal
0/500/1000		4	10%	230	192x192	R>L	130	5	110	7000-9000	DWI axial

Statistical analysis

Statistical analysis was performed using the statistical software package SPSS version 25 (IBM Corp., Armonk, NY). The chi-square test was used to analyze the relationship between pathological findings and patient characteristics. Also, a p-value of <0.05 was used to indicate statistical significance. Moreover, the presence of each pathological finding was analyzed in relation to patient age (≤35, 36-50, and >50 years) and gender.

## Results

In the study, 210 cases underwent an MRI of the brain to evaluate clinically suspected brain disorders, 138 (65.7 %) males and 72 (34.3%) females. The commonest pathological finding was mucosal thickening (111 cases, 52.9%). Retention cysts were present in around 14.3%, meaning 30 cases, and partial opacification was present in around 10%, representing 21 cases. Total opacification was present in three (1.4%) cases. Also, ostiomeatal complex obliteration was present in three (1.4 %) cases (Table 2). Mucosal thickening, partial opacification, total opacification, and ostiomeatal complex obliteration were more in males, whereas retention cysts were equal in both genders. A significant difference was found between gender and the presence of a retention cyst, with a p-value of 0.042 (Table 3). The incidence of paranal sinuses pathological findings on brain MRI categorized by age is shown in Table 4. The study showed that the paranasal pathological findings were more frequent in those aged ≤ 35 years, with a number of 75 (35.7%) cases. At the same time, 72 (34.3 %) cases were between 36 and 50 years of age. Simultaneously, there were 21 (10%) cases of pathological findings in those above 50 years of age. Mucosal thickening and retention cysts showed highly significant differences with different ages, with a p-value of 0.000. Also, partial and total opacification showed significant differences with different ages, with a p-value of 0.007.

**Table 2:** Frequencies of pathological findings in paranasal sinuses.

Frequency	Mucosal thickening	Retention cyst	Partial opacification	Total opacification	Ostiomeatal complex obliteration
Absent	99	180	189	207	207
Present	111	30	21	3	3
Total number	210	210	210	210	210
Present percentage	52.9%	14.3%	10%	1.4%	1.4%

**Table 3:** Incidence of paranasal sinuses pathological findings categorized by sex.

Gender	Mucosal thickening	Retention cyst	Partial opacification	Total opacification	Ostiomeatal complex obliteration
Male	78	15	15	3	3
Female	33	15	6	0	0
P-value	0.092	0.042	0.370	0.370	0.282
Total number	111	30	21	3	3

**Table 4:** Incidence of paranasal sinuses pathological findings categorized by age.

Age (year)	Mucosal thickening	Retention cyst	Partial opacification	Total opacification	Ostiomeatal complex obliteration	Total number
≤ 35	39	15	15	3	3	75
36–50	54	12	6	0	0	72
>50	18	3	0	0	0	21
P-value	0.000	0.000	0.007	0.007	0.056	
Total number	111	30	21	3	3	

## Discussion

In order to assess the paranasal sinuses, various imaging modalities were used. One of these is CT, which is commonly used to visualize the structures in the nasal cavity. However, MRI can also be used to visualize the region's tissue [12]. According to Nazri et al., MRI is more sensitive than a CT scan to discover sinus mucosal pathologies [12]. A significant concern in the literature was raised about the high radiation dose from CT scans [12]. The study was conducted on 210 patients referred for MRI scans for various neurological clinical conditions. The study participants were all aged 20 to 71 years old. The age distribution was ≤35, 36-50, and >50 years. Female patients made up 34.3% of the study group, while 65.7% were men. The pathological findings of the study were categorized into five groups: mucosal thickness, retention cyst, partial opacification, total opacification, and ostiomeatal complex obliteration. Out of the 210 patients, 111 (52.9%) showed mucosal thickening, while 30 (14.3%) showed a retention cyst. Also, 21 (10%) patients had partial opacification in their sinuses, and the remaining three (1.4%) had total opacification and ostiomeatal complex obliteration. Most commonly, mucosal thickening was detected. However, total opacification and ostiomeatal complex obliteration were less frequent in patients. The second most common pathological finding was a retention cyst, followed by partial opacification. Some patients had more than one pathological finding, and some had sinus involvement at multiple sites.

A previous study by McNeill et al. did not find a statistical relationship between the clinical symptoms of paranasal and MRI findings [13]. According to Maharjan, there was no link between the abnormal pathology in the sinus cavities and the symptoms [1]. Also, Kilickesmez et al. showed that the various morphological abnormalities in paranasal sinuses, such as retention cysts and mucosal thickness, do not correlate with the symptoms [11]. Instead of relying on radiological findings, management decisions should be based on nasal history and endoscopy findings [13]. On the other hand, Nazri et al. study showed that MRI findings are commonly associated with the symptoms of paranasal pathology [12]. For that, further research is needed to determine their clinical significance. The main objective of the brain MRI was to detect brain pathology, not to investigate paranasal sinus pathology. Instead, it was to visualize the entire paranal sinus structure using different MRI sequences. In Hansen et al.’s study, the thickening of mucosa was present in around 36% of the patients, while in the current study, mucosal thickening was present in around 52.9% [10]. The mucosal thickening in the current study was significantly higher than those reported in other studies. The present study showed retention cysts in about 14.3%, while Tarp et al. reported retention cysts in 15% of cases, which is almost the same as the present study [14]. A previous study by Yousefi et al. showed that mucosal thickening was the most common abnormal finding, followed by retention cysts, which aligns with the present study [15]. The results of the current study are supported by previous studies that investigated the prevalence of various paranasal sinus pathology, which found that mucosal thickening was the most common finding, followed by the retention cysts [5,10]. A previous study by Nazri et al. showed no significant difference in the incidence rate between the different age groups or genders [12].

In contrast, the present study showed that paranasal pathological findings were more frequent in those aged ≤ 35 years, with 75 cases (35.7%), and lower in those aged 36-50 years and above 50. Regarding gender, the present study showed that mucosal thickening, partial opacification, total opacification, and ostiomeatal complex obliteration were more in males, whereas the retention cysts were equal in both genders. Significant differences with different ages in the mucosal thickening, retention cysts, partial opacification, and total opacification were noted. Also, a significant difference was noted between gender differences and the presence of a retention cyst, with a p-value of 0.042.

Some limitations of this study should be addressed. Firstly, patients were diagnosed by a single radiologist. Secondly, all patients in the study were adults (more than 19 years old). Thirdly, the inclusion and exclusion criteria of the study could have been more thorough. Also, the lack of a comprehensive physical examination is another limitation of this study. A new hypothesis could be performed in a different study comparing the incidental paranasal sinus pathology in the pediatric and adult populations. It is also suggested that future studies should focus on the relationship between imaging findings and clinical observations.

## Conclusions

Pathological paranasal sinuses findings are commonly found in brain MRI during neurological disorder evaluation. These findings are usually benign, and mucosal thickening is the commonest. Most pathological findings were observed in those aged ≤ 35, while lower cases were in those aged 36-50 and above 50. Most of the paranal sinus pathologies were seen in the male gender. Detecting pathological findings in paranasal sinuses helps diagnose lesions unrelated to the suspected neurological disease.
